# Effects of oily fish and its fatty acid intake on non-alcoholic fatty liver disease development among South Korean adults

**DOI:** 10.3389/fnut.2022.876909

**Published:** 2022-07-22

**Authors:** Li-Juan Tan, Sangah Shin

**Affiliations:** Department of Food and Nutrition, Chung-Ang University, Seoul, South Korea

**Keywords:** oily fish consumption, omega-3 fatty acid, non-alcoholic fatty liver disease – NAFLD, cohort study (or longitudinal study), South Korean adults

## Abstract

**Background:**

The benefits of fish fatty acid intake for non-alcoholic fatty liver disease (NAFLD) are rarely reported, although a previous study assessed the relationship between oily fish consumption and the prevalence of NAFLD.

**Aims:**

We investigated whether oily fish and fish-based monounsaturated fatty acids, polyunsaturated fatty acids, and omega-3 fatty acids affect the development of NAFLD in South Korean adults.

**Methods:**

In this large-scale cohort study, 44,139 participants of the Health Examinees study were selected for analysis after 5 years of follow-up. NAFLD is diagnosed with a non-invasive index, the fatty liver index. Using multivariable Cox proportional hazards models, adjusted for age, body mass index, total energy intake, education, physical activity, smoking status, and drinking (alcohol) status, we calculated the hazard ratios and 95% confidence intervals.

**Results:**

For men, NAFLD had no statistically significant associations with quartiles of total oily fish or its fatty acid intake. However, among women, an inverse association was observed (all *p* for trend <0.05). Regarding the standard deviation (SD) increment of total oily fish or its fatty acid intake by one, all fatty acids from oily fish showed inverse associations for NAFLD in both men and women. After stratified analyses, we found that drinking status and menopause status were independent risk factors for NAFLD. Oily fish or its fatty acid intake has the same benefit pattern on metabolic dysfunction-associated fatty liver disease as NAFLD.

**Conclusion:**

Oily fish and its fatty acid intake showed a preventative benefit for NAFLD and metabolic dysfunction-associated fatty liver disease, especially in South Korean women.

## Introduction

Non-alcoholic fatty liver disease (NAFLD) is a condition characterized by predominant macro-vesicular steatosis of the liver, progressing from insulin resistance, and it can lead to steatohepatitis, fibrosis, or cirrhosis (1–3). NAFLD has become a major worldwide public health concern due to the increased risk of chronic diseases, such as type 2 diabetes mellitus and cardiovascular disease (4, 5). In the Western countries, nearly 25% of adults suffer from NAFLD, and this condition will become the most frequent indication of the need for liver transplantation by 2030 (4, 6). The overall prevalence of NAFLD is approximately 30% among South Korean adults and is twice as high in men as in women (7).

As per the existing knowledge, there are no specific drugs or therapeutic methods against NAFLD, although lifestyle (physical activity) and diet or nutrition management appear to be mainly responsible for preventing and treating NAFLD (8–10). Findings from the previous studies confirmed that a high fructose diet or a high-fat diet could accelerate NAFLD development (8, 11, 12). Fructose consumption increases insulin resistance and visceral fat and affects the lipoprotein lipase activity, leading to increased lipid uptake into the hypertrophied adipocytes (12, 13). Similarly, a high-fat diet, especially one rich in trans-fatty acid, leads to NAFLD by inducing obesity and insulin resistance (11, 14). However, not all fat in the diet is harmful to the liver; some dietary fatty acids, such as monounsaturated fatty acids (MUFAs) and polyunsaturated fatty acids (PUFAs), are considered to be beneficial to the liver (15). MUFAs and PUFAs can reduce lipid accumulation in the liver by affecting the activity of antioxidative enzymes (16). Oily fishes are a good source of MUFAs and PUFAs, especially omega-3 PUFAs. The most frequently consumed oily fishes in South Korea are mackerel, Pacific saury, and Spanish mackerel (17, 18).

This study aimed to investigate whether the intake of oily fish or its fatty acids affected NAFLD development among general South Korean adults, focusing on fish-based MUFAs, PUFAs, and omega-3 fatty acids. Additionally, we examined differences based on sex, age, body mass index (BMI), smoking status, drinking (alcohol) status, and menopausal status.

## Materials and methods

### Study population

The Health Examinees study is a large-scale prospective cohort study investigating epidemiologic characteristics, genomic features, and gene–environment interactions of major chronic diseases, such as cancer, in South Korea (19). The study protocol has been described in detail elsewhere (19). The baseline survey was conducted among adults aged 40–69 years between 2004 and 2013, and the first follow-up survey was initiated between 2012 and 2016 (*N* = 65,642). Among these participants, those with liver-related diseases (fatty liver disease, acute liver disease, chronic hepatitis, cirrhosis, cholelithiasis, cholecystitis, and thyroid disease) at baseline (*n* = 10,268), with missing outcome measures (blood biomarkers) (*n* = 5517), without sensible dietary information (energy intake <800/≥4,000 kcal/day for men and <500/≥3,500 kcal/day for women) (*n* = 859), with alcohol abuse (alcohol intake >210 g/week for men and >140 g/week for women) (*n* = 4824), with missing dietary information (*n* = 484), or with implausible BMI value (*n* = 35) were excluded in the current analysis, resulting in a final sample of 43,655 adults (women, 73.26%) (20–22). Detailed information on participant selection is shown in Supplementary Figure 1.

All participants voluntarily signed an informed written consent form before enrollment. This study was performed in accordance with the guidelines specified in the Declaration of Helsinki, and the study protocol was approved by the local Institutional Review Board (IRB) of the Ethics Committee of the Korean Genome and Epidemiology Study of the Korea National Institute of Health (IRB No. E-1503-103-657).

### Dietary assessment

Dietary data were collected using a 106-item semi-quantitative food frequency questionnaire (FFQ) developed for estimating food and nutrient consumption in South Korea. The reliability and validity of this questionnaire for South Koreans were established in a previous study assessed by four 3-day dietary records over four seasons (23). The dietary missing values were processed by imputation methods. Participants were asked about the average quantity and frequency of oily fish (mackerel/Pacific saury/Spanish mackerel) consumption during the past year at the survey time. Nine responses for frequency were ranged from “never or less than once per month” to “three times per day.” Moreover, the average portion sizes were estimated by photographs.

### Estimation of fatty acid intake

Data on each food item’s fatty acid content were obtained from the Korean Food Composition database 9.3 (24). First, food items of the FFQ containing more than one food component were separated according to their consumption ratios in each FFQ item, and consumption of each food item was converted into grams by multiplying each FFQ item’s daily consumption. Subsequently, daily fatty acid intake was calculated.

### Definition of non-alcoholic fatty liver disease

Non-alcoholic fatty liver disease was identified using the fatty liver index (FLI), a well-established non-invasive method to rule out patients with NAFLD (21, 25, 26). The FLI was calculated according to the following formula: FLI = 1/[1 + exp (−*x*)] × 100, *x* = 0.953 × ln (triglycerides) (mg/dl) + 0.139 × BMI (kg/m^2^) + 0.718 × ln (γ-glutamyl transferase) (U/L) + 0.053 × waist circumference (cm) − 15.745. The cut-off value for non-FLI-NAFLD was set to 30 (25–27).

For each participant, fasting venous blood was collected and processed by professionals (19). Weight (kg) and height (m) were also measured at the survey time. BMI was calculated as weight divided by the square of height.

### Statistical analyses

All analyses were stratified by sex and performed using SAS 9.4 (SAS Institute, Cary, NC, United States). Participants were divided into quartiles based on their intake of total oily fish and its fatty acid. Q1 represented the lowest consumption group, and Q4 represented the highest consumption group. General characteristics are presented as means and standard deviations (SDs) for continuous variables and frequencies (*n*, %) for categorical variables across quartiles of oily fish or its fatty acid intake. Differences between categories were tested by the general linear model for continuous variables and the Chi-square test for categorical variables.

Linear trends across the quartiles were tested by assigning each participant the median of the category and modeling this value as a continuous variable in models. Multivariable Cox proportional hazards models were performed to assess the relationship between FLI-NAFLD and oily fish or fatty acid ratio consumption. Results from the models were presented as hazard ratios (HRs) and 95% confidence intervals (CIs). The proportional hazard assumption was tested by including time-dependent covariates in the Cox model (*P* = 0.5116).

Sociodemographic and lifestyle characteristics, such as age, sex, level of education, physical activity, smoking, and alcohol drinking habits, were collected using standardized questionnaires. Educational level was categorized as low (under middle school), medium (high school), and high (college and above). Physical activity was determined based on participants’ participation in any sports, to the point of sweating for over 30 min, at least twice a week (21). Individuals were categorized according to their smoking status as a non-smoker, past-smoker, and current smoker. Similarly, they were categorized based on drinking status as non-drinker and current-drinker after excluding alcohol abusers.

Stratified analyses were also performed to test whether the associations relied on the confounder of interest. In the stratified analysis, multivariate Cox models (adjusted for continuous and categorical confounders) were applied to assess the association between the highest consumption quartile and FLI-NAFLD, separately for age categories (age < or ≥ median of age, 56-years for men and 52-years for women), BMI categories (BMI < 25 kg/m^2^ or BMI ≥ 25 kg/m^2^), smoking status (non-smoker, past-smoker, and current-smoker), drinking status (non-drinker and current-drinker), and menopause status (yes or no). For male participants, the analyses were stratified by age, BMI, smoking status, and drinking status, respectively, while in the case of female participants, menopausal status was added other than the aforementioned interests. Substitution analysis used the leave-one-out to calculate the associations with NAFLD by substituting certain fatty acid for another type (28).

Model 1 was adjusted for categorical confounders (education, physical activity, smoking status, and drinking status) and continuous variables (age, BMI, and total energy intake). Model 2 was adjusted for the same covariates as model 1 except for altering BMI to waist circumference. Model 3 was adjusted for the same covariates as model 1 plus energy percent from daily carbohydrate, protein, and fat intake. The statistical significance was set at *P* ≤ 0.05.

## Results

Baseline general characteristics and NAFLD incidence of the 43,655 participants included in the analysis across the quartiles of oily fish are given in Table 1 and Supplementary Table 1. Compared with the lowest consumption group (Q1), the highest consumption group (Q4) had lower age and higher waist circumference, higher BMI level, high educational level, higher physical activity, and were current-drinkers with higher alcohol consumption. Moreover, in terms of blood biomarkers, Q4 showed a lower level of serum triglycerides (all *P*-values < 0.05).

**TABLE 1:** Baseline general characteristics according to quartiles of oily fish consumption.

	Total	Q1	Q2	Q3	Q4	*P*-value
Men	11,672	1,137 (38.39%)	995 (38.06%)	1,213 (37.97%)	1,162 (40.06%)	
Age, years	55.65 ± 8.49	56.07 ± 8.65	55.27 ± 8.48	55.56 ± 8.40	55.64 ± 8.42	**0.0300**
BMI, kg/m^2^	23.74 ± 2.26	23.59 ± 2.32	23.70 ± 2.28	23.76 ± 2.22	23.89 ± 2.22	**<0.0001**
Waist circumference, cm	83.76 ± 6.51	83.28 ± 6.72	83.64 ± 6.54	84.00 ± 6.40	84.11 ± 6.37	**<0.0001**
Smoking status						0.4173
Non-smoker	4,097 (35.10%)	1,069 (36.09%)	937 (35.85%)	1,109 (34.71%)	982 (33.85%)	
Past-smoker	4,908 (42.05%)	1,239 (41.83%)	1,063 (40.67%)	1,359 (42.54%)	1,247 (42.99%)	
Current-smoker	2,667 (22.85%)	654 (22.08%)	614 (23.49%)	727 (22.75%)	672 (23.16%)	
Educational level						**<0.0001**
Under middle school	2,388 (20.63%)	743 (25.39%)	548 (21.13%)	626 (19.76%)	471 (16.33%)	
High school	4,575 (39.53%)	1,161 (39.68%)	1,030 (39.71%)	1,260 (39.77%)	1,124 (38.96%)	
College or above	4,610 (39.83%)	1,022 (34.93%)	1,016 (39.17%)	1,282 (40.47%)	1,290 (44.71%)	
Physical activity						**<0.0001**
Inactive	8,864 (77.77%)	2,332 (80.22%)	1,998 (78.17%)	2,469 (79.16%)	2,065 (73.33%)	
Active	2,534 (22.23%)	575 (19.78%)	558 (21.83%)	650 (20.84%)	751 (26.67%)	
Drinking status						**<0.0001**
Non-drinker	3,997 (34.24%)	1,154 (38.96%)	886 (33.89%)	1,034 (32.36%)	923 (31.82%)	
Current-drinker	7,675 (65.76%)	1,808 (61.04%)	1,728 (66.11%)	2,161 (67.64%)	1,978 (68.18%)	
Alcohol consumption, g/day	6.31 ± 7.89	5.45 ± 7.48	6.49 ± 8.09	6.61 ± 8.01	6.69 ± 7.92	**0.0013**
Total energy intake, kcal/day	1,831.96 ± 534.95	1,672.17 ± 500.07	1,751.89 ± 500.90	1,867.44 ± 482.14	2,028.19 ± 585.25	**<0.0001**
Carbohydrate, E%	72.86 ± 7.00	75.04 ± 6.54	73.12 ± 6.38	71.65 ± 6.88	69.52 ± 7.31	**<0.0001**
Protein, E%	13.29 ± 2.37	12.21 ± 1.87	12.89 ± 1.87	13.41 ± 2.09	14.30 ± 2.44	**<0.0001**
Fat, E%	13.85 ± 5.10	12.75 ± 5.06	13.99 ± 4.90	14.94 ± 5.18	16.18 ± 5.31	**<0.0001**
Oily fish intake, g/day	6.30 ± 6.17	1.50 ± 0.73	3.47 ± 0.62	6.08 ± 0.92	14.01 ± 7.81	**<0.0001**
FLI	27.73 ± 18.07	27.52 ± 18.06	27.76 ± 18.29	27.58 ± 17.99	28.07 ± 17.98	0.7764
AST, IU/L	23.56 ± 10.81	23.07 ± 6.97	23.59 ± 8.61	23.59 ± 8.25	23.97 ± 16.68	**0.0164**
ALT, IU/L	23.35 ± 12.38	22.80 ± 11.70	23.30 ± 12.32	23.34 ± 11.71	23.97 ± 13.74	**0.0019**
γ-GTP, IU/L	31.04 ± 22.79	30.39 ± 23.23	31.43 ± 25.00	31.10 ± 21.57	31.29 ± 21.52	0.6248
TG, mg/dL	120.70 ± 61.45	124.60 ± 64.02	120.84 ± 61.42	118.82 ± 60.60	118.64 ± 59.54	**0.0002**
Women	31,983	1,614 (18.71%)	1,254 (18.72%)	1,563 (18.75%)	1,543 (18.54%)	
Age, years	52.69 ± 7.67	53.24 ± 7.94	52.24 ± 7.69	52.53 ± 7.63	52.63 ± 7.38	**<0.0001**
BMI, kg/m^2^	23.32 ± 2.62	23.22 ± 2.63	23.28 ± 2.64	23.34 ± 2.62	23.43 ± 2.59	**<0.0001**
Waist circumference, cm	77.40 ± 7.47	77.23 ± 7.57	77.25 ± 7.49	77.47 ± 7.39	77.61 ± 7.42	**<0.0001**
Smoking status						0.3490
Non-smoker	31,293 (97.84%)	8,429 (97.72%)	6,544 (97.72%)	8,170 (98.01%)	8,150 (97.91%)	
Past-smoker	279 (0.87%)	75 (0.87%)	58 (0.87%)	64 (0.77%)	82 (0.99%)	
Current-smoker	411 (1.29%)	122 (1.41%)	95 (1.42%)	102 (1.22%)	92 (1.11%)	
Educational level						**<0.0001**
Under middle school	11,116 (35.10%)	3,493 (40.90%)	2,376 (35.80%)	2,783 (33.75%)	2,464 (29.88%)	
High school	14,073 (44.44%)	3,502 (41.00%)	2,884 (43.45%)	3,775 (45.79%)	3,912 (47.44%)	
College or above	6,481 (20.46%)	1,546 (18.10%)	1,377 (20.75%)	1,687 (20.46%)	1,871 (22.69%)	
Physical activity						**<0.0001**
Inactive	25,342 (80.83%)	6,992 (82.42%)	5,381 (81.46%)	6,653 (81.47%)	6,316 (78.00%)	
Active	6,010 (19.17%)	1,491 (17.58%)	1,225 (18.54%)	1,513 (18.53%)	1,781 (22.00%)	
Drinking status						**0.0010**
Non-drinker	23,035 (72.02%)	6,345 (73.56%)	4,755 (71.00%)	5,936 (71.21%)	5,999 (72.07%)	
Current-drinker	8,948 (27.98%)	2,281 (26.44%)	1,942 (29.00%)	2,400 (28.79%)	2,325 (27.93%)	
Alcohol consumption, g/day	0.97 ± 2.62	0.89 ± 2.49	1.02 ± 2.73	1.00 ± 2.63	0.97 ± 2.67	0.5169
Total energy intake, kcal/day	1,701.87 ± 566.57	1,537.54 ± 494.25	1,621.75 ± 505.69	1,716.52 ± 516.94	1,921.95 ± 652.05	**<0.0001**
Carbohydrate, E%	72.83 ± 7.42	74.21 ± 7.20	72.46 ± 7.15	71.32 ± 7.24	69.04 ± 7.62	**<0.0001**
Protein, E%	13.52 ± 2.53	12.61 ± 2.12	13.25 ± 2.15	13.67 ± 2.22	14.58 ± 2.53	**<0.0001**
Fat, E%	13.65 ± 5.37	13.18 ± 5.49	14.28 ± 5.39	15.00 ± 5.41	16.38 ± 5.56	**<0.0001**
Oily fish intake, g/day	6.48 ± 6.80	1.37 ± 0.77	3.49 ± 0.65	6.11 ± 0.92	14.54 ± 8.75	**<0.0001**
FLI	17.89 ± 15.48	17.98 ± 15.55	17.84 ± 15.45	17.84 ± 15.52	17.89 ± 15.41	0.9001
AST, IU/L	22.04 ± 10.55	22.16 ± 14.85	21.95 ± 9.11	21.93 ± 8.27	22.09 ± 8.01	0.6095
ALT, IU/L	18.93 ± 14.06	18.96 ± 16.88	18.69 ± 12.81	18.92 ± 13.61	19.10 ± 12.10	0.3479
γ-GTP, IU/L	19.97 ± 15.24	20.08 ± 15.30	19.77 ± 15.60	19.90 ± 14.70	20.09 ± 15.39	0.4464
TG, mg/dL	107.3 ± 62.11	113.41 ± 67.62	106.62 ± 61.29	106.14 ± 59.57	102.66 ± 58.72	**<0.0001**

AST, aspartate transaminase; ALT, alanine aminotransferase; BMI, body mass index; FLI, fatty liver index; NAFLD, non-alcoholic fatty liver disease; Q, quartile; TG, triglyceride; WC, waist circumference; γ-GTP, γ-glutamyl transpeptidase. Values are shown as means ± standard deviations or n (%).

Comparisons were performed using a Chi-square test for categorical variables and a generalized linear model for continuous variables. Boldface indicates statistical significance (P < 0.05).

FLI = 1/[1 + exp(−x)] × 100, x = 0.953 × ln (TG) (mg/dL) + 0.139 × BMI (kg/m^2^) + 0.718 × ln (γ-GTP) (U/L) + 0.053 × WC (cm) − 15.745; (cut-off value, 30).

The 1-SD increment analysis of the association between FLI-NAFLD and oily fish and its fatty acid intake revealed an inverse association among female participants. The HRs of oily fish intake, ratio of total fatty acid from oily fish and total daily diet, ratio of MUFA from oily fish and total daily diet, ratio of PUFA, and ratio of omega-3 fatty acid were 0.935 (95% CI: 0.910–0.961), 0.927 (95% CI: 0.903–0.951), 0.934 (95% CI: 0.910–0.958), 0.935 (95% CI: 0.911–0.959), and 0.935 (95% CI: 0.912–0.959), respectively (Table 2). However, the association between oily fish consumption and FLI-NAFLD was absent for male participants (Table 2).

**TABLE 2:** Hazard ratios (HRs) for fatty liver index – non-alcoholic fatty liver disease (FLI-NAFLD) according to the quartiles of oily fish and its fatty acid intake.

	Q1	Q2	Q3	Q4	*P* for trend	1-SD increment
**Men**						
Oily fish	Ref	0.898 (0.824, 0.978)	0.958 (0.883, 1.040)	0.965 (0.886, 1.050)	0.9399	0.974 (0.946, 1.002)
Total fatty acid/fat	Ref	0.974 (0.898, 1.058)	0.941 (0.866, 1.022)	0.901 (0.828, 0.981)	**0.0116**	0.942 (0.911, 0.973)
MUFA/total dietary MUFA	Ref	0.947 (0.872, 1.028)	0.942 (0.867, 1.023)	0.947 (0.872, 1.030)	0.3524	0.957 (0.926, 0.989)
PUFA/total dietary PUFA	Ref	0.997 (0.918, 1.083)	0.983 (0.905, 1.068)	0.976 (0.898, 1.061)	0.5293	0.964 (0.935, 0.994)
Omega-3/dietary omega-3	Ref	0.993 (0.914, 1.079)	1.017 (0.936, 1.104)	0.956 (0.879, 1.038)	0.2831	0.962 (0.934, 0.990)
**Women**						
Oily fish	Ref	0.925 (0.859, 0.996)	0.962 (0.897, 1.032)	0.839 (0.780, 0.902)	**<0.0001**	0.935 (0.910, 0.961)
Total fatty acid/fat	Ref	0.890 (0.828, 0.957)	0.878 (0.817, 0.944)	0.806 (0.750, 0.867)	**<0.0001**	0.927 (0.903, 0.951)
MUFA/total dietary MUFA	Ref	0.918 (0.853, 0.988)	0.907 (0.843, 0.975)	0.834 (0.776, 0.896)	**<0.0001**	0.934 (0.910, 0.958)
PUFA/total dietary PUFA	Ref	0.953 (0.886, 1.025)	0.916 (0.853, 0.985)	0.858 (0.799, 0.922)	**<0.0001**	0.935 (0.911, 0.959)
Omega-3/dietary omega-3	Ref	0.939 (0.873, 1.009)	0.948 (0.882, 1.018)	0.853 (0.794, 0.917)	**<0.0001**	0.935 (0.912, 0.959)

BMI, body mass index; FLI, fatty liver index; HRs, hazard ratios; MUFA, monounsaturated fatty acid; NAFLD, non-alcoholic fatty liver disease; PUFA, polyunsaturated fatty acids; Q, quartile; SD, standard deviation; WC, waist circumference. Oily fish: Mackerel/Pacific saury/Spanish mackerel. Total fatty acid/fat: ratio of total fatty acid from oily fish and total dietary fatty acid. MUFA/total dietary MUFA: ratio of total MUFA from oily fish and total dietary fatty acid. PUFA/total dietary PUFA: ratio of total PUFA from oily fish and total dietary fatty acid. Omega-3/dietary omega-3: ratio of total omega-3 PUFA from oily fish and total dietary fatty acid. Model was adjusted by age, BMI, total energy intake, smoking status, drinking status, physical activity level, and educational level. Boldface indicates statistical significance (P < 0.05). NAFLD is defined by an FLI of >30. FLI = 1/[1 + exp(−x)] × 100, x = 0.953 × ln (TG) (mg/dL) + 0.139 × BMI (kg/m^2^) + 0.718 × ln (γ-GTP) (U/L) + 0.053 × WC (cm) − 15.745.

The quartile analysis revealed that female participants consuming higher quantities of oily fish were less likely to have FLI-NAFLD (highest *vs*. lowest quartile, HR: 0.839; 95% CI: 0.780–0.902; *P* for trend <0.05; Table 2). The multivariate-adjusted analysis also revealed that the highest quartile of fatty acid ratio was significantly associated with FLI-NAFLD (Table 2). However, these significances were only present in female participants, except that the association with the total fatty acid ratio was significant in both sexes (highest vs. lowest quartile, HR: 0.901; 95% CI: 0.828–0.981; HR: 0.806; 95% CI: 0.750–0.867 in male and female participants, respectively, both *P* for trend <0.05; Table 2). After further adjusted analyses (models 2 and 3, Supplementary Table 2), the results stayed consistent.

**FIGURE 1:**
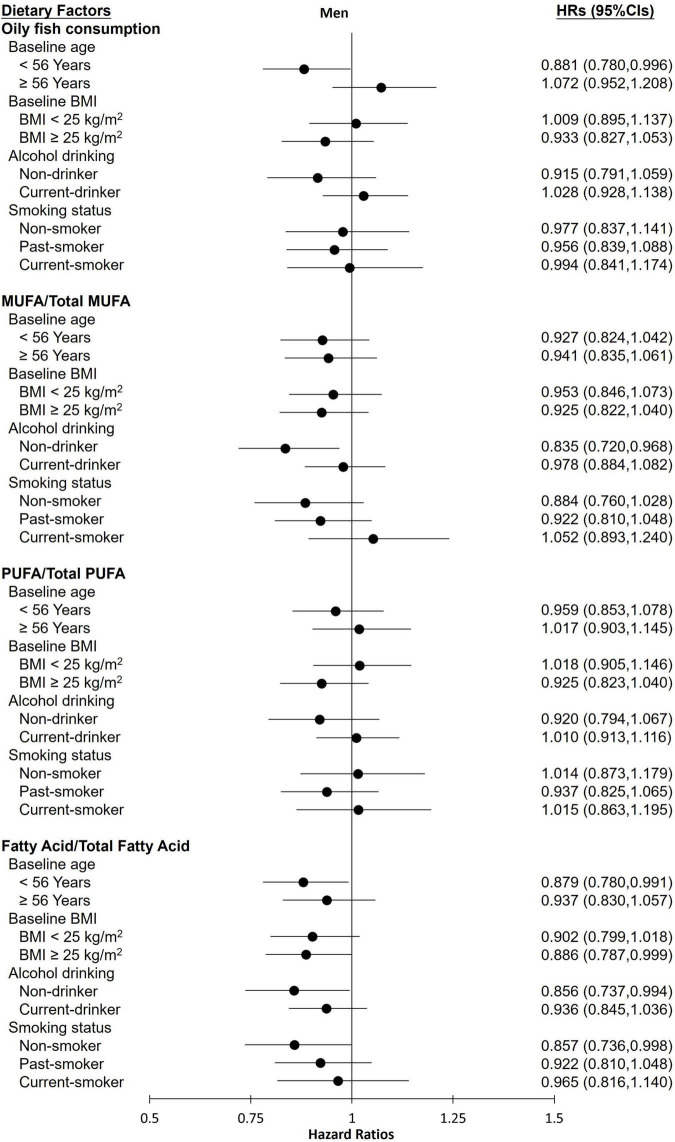
Hazard ratios (HRs) of fatty liver index (FLI)-non-alcoholic fatty liver disease for the highest categories compared with the lowest categories of oily fish and its fatty acid intake among male participants in the current cohort study. Analyses were stratified by body mass index (BMI), age, smoking status, and drinking status. HRs, hazard ratios; MUFAs, monounsaturated fatty acids; PUFAs, polyunsaturated fatty acids.

**FIGURE 2:**
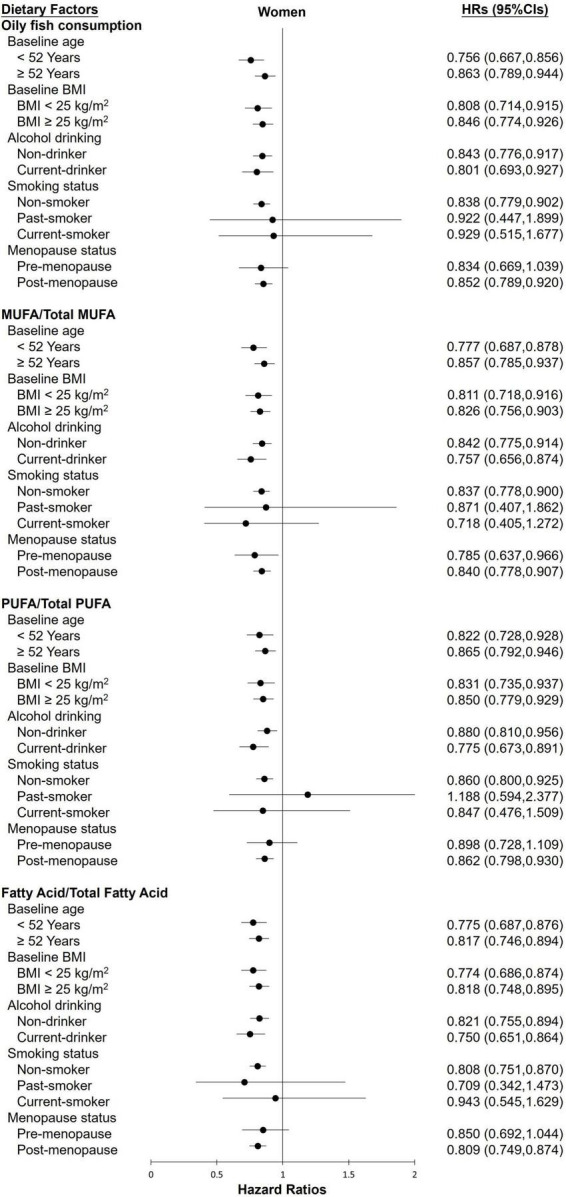
The HRs of FLI-non-alcoholic fatty liver disease for the highest categories compared with the lowest categories of oily fish and its fatty acid intake among female participants in the current cohort study. Analyses were stratified by body mass index (BMI), age, smoking status, drinking status, and menopause status. HRs, hazard ratios; MUFAs, monounsaturated fatty acids; PUFAs, polyunsaturated fatty acids.

The stratified analysis of participants revealed almost no differences in the effect of various groups toward FLI-NAFLD (Figures 1, 2). Among female participants, the association between the various dietary exposure groups and FLI-NAFLD was maintained regardless of age, BMI, and drinking status, whereas smoking status and menopause status were weakly associated with FLI-NAFLD (Figure 2). Among non-smokers and post-menopausal participants, oily fish or its fatty acid intake resulted in a significantly lower risk of FLI-NAFLD development (Figure 2). Age and drinking status were weakly associated with FLI-NAFLD among male participants (Figure 1).

The results from a leave-one-out substitution analysis are shown in Supplementary Table 3. Among female participants, after replacing fatty acid intake (from other food sources) with a fatty acid intake predominantly from oily fish, a one-unit increment of fatty acid was associated with a lower risk of FLI-NAFLD. The converse was also true. All adjusted models showed the same association pattern. However, there was no significant association for male participants.

## Discussion

Here, we evaluated the association between oily fish intake and its fatty acid consumption and FLI-defined NAFLD in a large-scale cohort of general adults recruited from 38 sites of South Korea.

A significant inverse association between high oily fish intake and FLI-NAFLD among female participants was found. The analysis also revealed that oily fish-sourced fatty acids, such as MUFAs, PUFAs, and omega-3 PUFAs, have preventative benefits for NAFLD. Moreover, the association continued to exist after being stratified by age, BMI, smoking status, drinking status, and menopausal status. Although covariates, such as smoking status and menopausal status, impact the effects of oily fish and its fatty acid intake on FLI-NAFLD after stratified analysis, in most ways, total intake of oily fish and its fatty acids resulted in preventative effects independently, regardless of age, BMI, and drinking alcohol status.

Non-alcoholic fatty liver disease is a common chronic disease wherein triglycerides accumulate excessively in the liver without alcohol abuse (29). Moreover, NAFLD is closely associated with diabetes and metabolic syndrome, which are both related to the pathophysiology of inflammation and insulin resistance (30, 31). Dietary MUFAs have been reported to improve lipid profile through their anti-inflammatory characteristics (30, 32). Oily fish is protective against NAFLD owing to its omega-3 PUFA contents, which impact the lipid profile (33–35). Mackerel and Pacific saury are the types of oily fishes that have been reported to be enriched in various fatty acids, especially omega-3 PUFA (36–40).

Omega-3 PUFA has been previously associated with reducing the risk of NAFLD development in various epidemiological studies (41–46). A dietary intervention study suggested that patients with NAFLD showed a lower level of circulating liver enzymes and triglycerides, with a significant improvement of adiponectin after long-term (1 year) consumption of omega-3 PUFA (42). Furthermore, a cross-sectional study revealed that fish and omega-3 PUFA intake was associated with decreasing portal and lobular inflammation and a lower risk for hepatic inflammation among children (43). In Japan, a cross-sectional study conducted in adults showed that omega-3 PUFA was not an independent risk factor for NAFLD. However, dietary eicosapentaenoic acid and eicosapentaenoic acid + docosahexaenoic acid were preventive nutrients for NAFLD and improved inflammatory change in adipose tissue in men (47, 48). These findings are partially in line with our results that oily fish and its omega-3 PUFA content are associated with a lower incidence of FLI-NAFLD, while the effect of omega-6 PUFA content has not yet been fully elucidated.

Our findings also suggest that menopausal status is an independent risk factor for FLI-NAFLD among female participants. This result is partially in line with that of a previous study, which demonstrated that menopausal status change was correlated with NAFLD through altering sex hormones, and dietary factors could exacerbate the relationship (29). The previous study convinced that smoking and drinking were associated with higher prevalence of NAFLD (49) and in current study, men and women also showed gender differences in smoking and drinking habits shown in Table 1. Less current smokers and alcohol drinkers in women than men participants maybe another explanation to significant results only found in women. Further, it is important to consider sex difference, which may be another risk factor leading to different results of this study. A previous review research reported that adipose tissue distribution, gut microbiota, and innate immune response showed some sex differences (50). Adipose tissue and innate immune response play an important role in regulating insulin resistance and inflammatory reaction (51, 52). The gut microbiota could regulate lipid/glucose metabolism by activating the farnesoid X receptor (53).

The strength of the current study is that we conducted a large-scale cohort study in South Korea, and the result is partly adapted to the general population. Moreover, in PUFA and MUFA, we focused on oily fish-sourced fatty acids instead of its supplements or other dietary sources. However, this study has certain limitations. First, the diagnosis of NAFLD was not based on a liver biopsy. However, the FLI used in the current study has been evaluated and verified in a previous study and is considered an appropriate tool for large nutritional epidemiological studies (21, 25, 26, 54). Second, rather than the exact time that FLI-NAFLD occurred, the endpoint was set on the time conducting follow-up survey. Considering soft endpoints more common in observational study, and it has little impacts on large-scale cohort study, this limitation could be negligible (55). Third, the fish-sourced fatty acid contents were not measured directly, but through linking the Korean Food Composition Database 9.3 to FFQ data, and different cooking or storage methods may lead to possible bias. Future studies should consider these factors. Finally, the lifestyle of participants was assessed by a self-reported questionnaire such as smoking, drinking, and physical activity, which may be overreported or under reported. So we grouped them as categorical variables when adjusting model to minimize the reporting bias.

## Conclusion

In conclusion, we have demonstrated that the intake of oily fish and its fatty acid contents, such as MUFAs, PUFAs, and omega-3 PUFAs, is associated with a lower incidence of NAFLD. As a result, although we did not study their precise molecular mechanisms, oily fish may be considered effective preventative strategies for NAFLD development among South Koreans, especially for women. These findings may provide a basis for revising middle-aged and older adults’ dietary guidelines in South Korea.

## Data availability statement

The datasets presented in this article are not readily available because the datasets analyzed for this study can be available from National Genome Research Institute, Korea Centers for Disease Control and Prevention. Restrictions apply to the availability of these data, which were used under license for this study. Data described in the manuscript, codebook, and analytic code are available from the authors with the permission of National Genome Research Institute, Korea Centers for Disease Control and Prevention. Requests to access the datasets should be directed to National Genome Research Institute, Korea Centers for Disease Control and Prevention; https://kdca.go.kr/contents.es?mid=a40504060100.

## Ethics statement

The studies involving human participants were reviewed and approved by the Institutional Review Board (IRB) of the Ethics Committee of the Korean Genome and Epidemiology Study of the Korea National Institute of Health (IRB No. E-1503-103-657). The patients/participants provided their written informed consent to participate in this study.

## Authors contributions

SS designed and conducted the research and reviewed and revised the manuscript critically. L-JT analyzed the data, performed the statistical analysis, and wrote the first draft of the manuscript. SS and L-JT had primary responsibility for the final content. Both authors approved the final version of the article.

## Conflict of interest

The authors declare that the research was conducted in the absence of any commercial or financial relationships that could be construed as a potential conflict of interest.

## Publisher’s note

All claims expressed in this article are solely those of the authors and do not necessarily represent those of their affiliated organizations, or those of the publisher, the editors and the reviewers. Any product that may be evaluated in this article, or claim that may be made by its manufacturer, is not guaranteed or endorsed by the publisher.

## Abbreviations

ALT: alanine aminotransferase
BMI: body mass index
CIs: confidence intervals
FFQ: food frequency questionnaire
FLI: fatty liver index
HRs: hazard ratios
MUFAs: monounsaturated fatty acids
NAFLD: non-alcoholic fatty liver disease
PUFAs: polyunsaturated fatty acids
SDs: standard deviations.
