# Supplementary material for: Effects of oily fish and its fatty acid intake on non-alcoholic fatty liver disease development among South Korean adults
Source: Front Nutr. 2022 Jul 22;9:876909. doi: 10.3389/fnut.2022.876909 (PMC9353947; doi:10.3389/fnut.2022.876909)
Supplement: Supplementary file 1 [file Data_Sheet_1.docx]

Supplementary Material

# Supplementary and Tables

Supplementary Table 1. Incidence of fatty liver index-non-alcoholic fatty liver disease according to quartiles of oily fish and its fatty acid intake

|  | Q1 | Q2 | Q3 | Q4 |
| --- | --- | --- | --- | --- |
|  | Cases (incidence) | | | |
| Men, n = 11,672 |  |  |  |  |
| Oily fish | 1137 (38.39%) | 995 (38.06%) | 1213 (37.97%) | 1162 (40.06%) |
| Total fatty acid/fat | 1137 (38.97%) | 1148 (39.34%) | 1118 (38.31%) | 1104 (37.83%) |
| MUFA/total dietary MUFA | 1131 (38.76%) | 1139 (39.03%) | 1126 (38.59%) | 1111 (38.07%) |
| PUFA/ total dietary PUFA | 1114 (38.18%) | 1145 (39.24%) | 1120 (38.38%) | 1128 (38.66%) |
| Omega-3/dietary omega-3 | 1113 (38.14%) | 1125 (38.55%) | 1147 (39.31%) | 1122 (38.45%) |
|  |  |  |  |  |
| Women, n = 31,983 |  |  |  |  |
| Oily fish | 1614 (18.71%) | 1254 (18.72%) | 1563 (18.75%) | 1543 (18.54%) |
| Total fatty acid/fat | 1469 (18.37%) | 1452 (18.16%) | 1483 (18.55%) | 1570 (19.63%) |
| MUFA/total dietary MUFA | 1418 (17.74%) | 1429 (17.87%) | 1509 (18.87%) | 1618 (20.24%) |
| PUFA/ total dietary PUFA | 1462 (18.29%) | 1450 (18.13%) | 1487 (18.60%) | 1575 (19.70%) |
| Omega-3/dietary omega-3 | 1488 (18.61%) | 1465 (18.32%) | 1501 (18.77%) | 1520 (19.01%) |

BMI, body mass index; FLI, fatty liver index; MUFA, monounsaturated fatty acid; NAFLD, non-alcoholic fatty liver disease, PUFA, polyunsaturated fatty acids.

Values are shown as cases (%).

Oily fish: Mackerel/Pacific saury/Spanish mackerel

Total fatty acid/fat: ratio of total fatty acid from oily fish and total dietary fatty acid

MUFA/total dietary MUFA: ratio of total MUFA from oily fish and total dietary fatty acid

PUFA/ total dietary PUFA: ratio of total PUFA from oily fish and total dietary fatty acid

Omega-3/dietary omega-3: ratio of total omega-3 PUFA from oily fish and total dietary fatty acid

NAFLD is defined by FLI index over than 30.

FLI = 1 / (1 + exp(-x)) × 100, x = 0.953 × ln (TG) (mg/dL) + 0.139 × BMI (kg/m^2^) + 0.718 × ln (γ-GTP) (U/L) + 0.053 × WC (cm) - 15.745.

Supplementary Table 2. HRs for FLI-NAFLD according to quartiles of oily fish and its fatty acid intake in models 2 and 3

|  | Q1 | Q2 | Q3 | Q4 | *P* for trend |
| --- | --- | --- | --- | --- | --- |
| Men |  |  |  |  |  |
| Oily fish |  |  |  |  |  |
| Model 2 | Ref | **0.899**  **(0.825, 0.979)** | 0.958  (0.882, 1.039) | 0.972  (0.893, 1.058) | 0.7927 |
| Model 3 | Ref | **0.890**  **(0.817, 0.970)** | 0.945  (0.870, 1.028) | 0.943  (0.862, 1.031) | 0.6934 |
| Total fatty acid/fat |  |  |  |  |  |
| Model 2 | Ref | 0.983  (0.905, 1.067) | 0.955  (0.879, 1.037) | **0.906**  **(0.833, 0.986)** | **0.0146** |
| Model 3 | Ref | 0.977  (0.899, 1.061) | 0.947  (0.870, 1.030) | **0.910**  **(0.833, 0.993)** | **0.0280** |
| MUFA/total dietary MUFA |  |  |  |  |  |
| Model 2 | Ref | 0.938  (0.863, 1.018) | 0.932  (0.858, 1.012) | 0.927  (0.852, 1.008) | 0.1548 |
| Model 3 | Ref | 0.950  (0.874, 1.032) | 0.948  (0.872, 1.030) | 0.954  (0.875, 1.041) | 0.4691 |
| PUFA/ total dietary PUFA |  |  |  |  |  |
| Model 2 | Ref | 0.991  (0.912, 1.076) | 0.986  (0.907, 1.071) | 0.962  (0.885, 1.045) | 0.3370 |
| Model 3 | Ref | 0.996  (0.917, 1.082) | 0.981  (0.902, 1.067) | 0.975  (0.895, 1.061) | 0.5147 |
| Omega-3/dietary omega-3 |  |  |  |  |  |
| Model 2 | Ref | 0.991  (0.912, 1.077) | 1.017  (0.936,  1.104) | 0.949  (0.873, 1.031) | 0.2116 |
| Model 3 | Ref | 0.991  (0.912, 1.077) | 1.012  (0.931, 1.099) | 0.951  (0.875, 1.034) | 0.2399 |
|  |  |  |  |  |  |
| Women |  |  |  |  |  |
| Oily fish |  |  |  |  |  |
| Model 2 | Ref | 0.956  (0.887, 1.029) | 0.989  (0.922, 1.061) | **0.874**  **(0.813, 0.940)** | **0.0003** |
| Model 3 | Ref | **0.918**  **(0.852, 0.990)** | 0.953  (0.887, 1.023) | **0.823**  **(0.762, 0.889)** | **< 0.0001** |
| Total fatty acid/fat |  |  |  |  |  |
| Model 2 | Ref | **0.904**  **(0.841, 0.973)** | **0.890**  **(0.828, 0.957)** | **0.827**  **(0.769, 0.889)** | **< 0.0001** |
| Model 3 | Ref | **0.898**  **(0.835, 0.966)** | **0.894**  **(0.831, 0.963)** | **0.829**  **(0.769, 0.894)** | **< 0.0001** |
| MUFA/total dietary MUFA |  |  |  |  |  |
| Model 2 | Ref | 0.946  (0.879, 1.018) | **0.902**  **(0.839, 0.970)** | **0.857**  **(0.798, 0.921)** | **< 0.0001** |
| Model 3 | Ref | **0.923**  **(0.858, 0.994)** | **0.919**  **(0.854, 0.989)** | **0.854**  **(0.793, 0.921)** | **0.0001** |
| PUFA/ total dietary PUFA |  |  |  |  |  |
| Model 2 | Ref | 0.948  (0.882, 1.020) | **0.917**  **(0.853, 0.985)** | **0.856**  **(0.798, 0.920)** | **< 0.0001** |
| Model 3 | Ref | 0.956  (0.888, 1.028) | **0.922**  **(0.857, 0.992)** | **0.867**  **(0.806, 0.934)** | **0.0001** |
| Omega-3/dietary omega-3 |  |  |  |  |  |
| Model 2 | Ref | 0.957  (0.891, 1.029) | 0.959  (0.892, 1.030) | **0.868**  **(0.808, 0.932)** | **< 0.0001** |
| Model 3 | Ref | 0.940  (0.875, 1.011) | 0.948  (0.882, 1.019) | **0.854**  **(0.794, 0.919)** | **< 0.0001** |

BMI, body mass index; FLI, fatty liver index; HRs, hazard ratios; MUFAs, monounsaturated fatty acids; NAFLD, non-alcoholic fatty liver disease; PUFAs, polyunsaturated fatty acids. Model 2 was adjusted for age, waist circumference, total energy intake, smoking status, drinking status, physical activity level, and educational level.

Model 3 was adjusted for age, BMI, total energy intake, smoking status, drinking status, physical activity level, educational level, and percent of energy from carbohydrate, protein, and fat.

Boldface indicates statistical significance (*P* < 0.05)

Supplementary Table 3. Hazard ratios for NAFLD derived from the multiple Cox model with one-unit increment of fatty acids in the HEXA population

|  | HR (95% CI) | | |
| --- | --- | --- | --- |
|  | Model 1 | Model 2 | Model 3 |
| Men |  |  |  |
| Total fatty acid |  |  |  |
| Fish for other foods | 0.987 (0.948, 1.029) | 1.002 (0.961, 1.045) | 0.982 (0.940, 1.026) |
| Other foods for fish | 1.018 (0.974, 1.063) | 0.998 (0.957, 1.040) | 1.013 (0.972, 1.055) |
| MUFA |  |  |  |
| Fish for other foods | 0.958 (0.865, 1.062) | 1.000 (0.906, 1.104) | 0.969 (0.879, 1.068) |
| Other foods for fish | 1.044 (0.942, 1.157) | 1.000 (0.906, 1.104) | 1.032 (0.936, 1.138) |
| PUFA |  |  |  |
| Fish for other foods | 0.936 (0.777, 1.127) | 1.014 (0.847, 1.212) | 0.962 (0.806, 1.148) |
| Other foods for fish | 1.069 (0.888, 1.287) | 0.987 (0.825, 1.180) | 1.039 (0.871, 1.241) |
|  |  |  |  |
| Women |  |  |  |
| Total fatty acid |  |  |  |
| Fish for other foods | **0.919 (0.888, 0.952)** | **0.933 (0.901, 0.966)** | **0.921 (0.887, 0.956)** |
| Other foods for fish | 1.086 (1.046, 1.127) | 1.072 (1.035, 1.109) | 1.088 (1.050, 1.126) |
| MUFA |  |  |  |
| Fish for other foods | **0.820 (0.751, 0.895)** | **0.853 (0.786, 0.924)** | **0.824 (0.759, 0.893)** |
| Other foods for fish | 1.220 (1.118, 1.332) | 1.173 (1.082, 1.272) | 1.214 (1.119, 1.317) |
| PUFA |  |  |  |
| Fish for other foods | **0.697 (0.595, 0.817)** | **0.731 (0.631, 0.847)** | **0.704 (0.607, 0.817)** |
| Other foods for fish | 1.435 (1.224, 1.682) | 1.368 (1.180, 1.585) | 1.420 (1.224, 1.648) |

BMI, body mass index; CIs, confidence intervals; HEXA, The Health Examinees study; HRs, hazard ratios; MUFA, monounsaturated fatty acid; NAFLD, non-alcoholic fatty liver disease, PUFA, polyunsaturated fatty acids.

Model 1 was adjusted for age, BMI, total energy intake, smoking status, drinking status, physical activity level, and educational level.

Model 2 was adjusted for age, waist circumference, total energy intake, smoking status, drinking status, physical activity level, and educational level.

Model 3 was adjusted for age, BMI, total energy intake, smoking status, drinking status, physical activity level, educational level, and percent of energy from carbohydrates, protein, and fat.

Boldface indicates statistical significance (*P* < 0.05).

Fish for other foods: one-unit increment of oily fish sourced fatty acid after replacing other food sourced fatty acid

Other foods for fish: one-unit increment of other food sourced fatty acid after replacing oily fish sourced fatty acid

**2 Supplementary Figures**


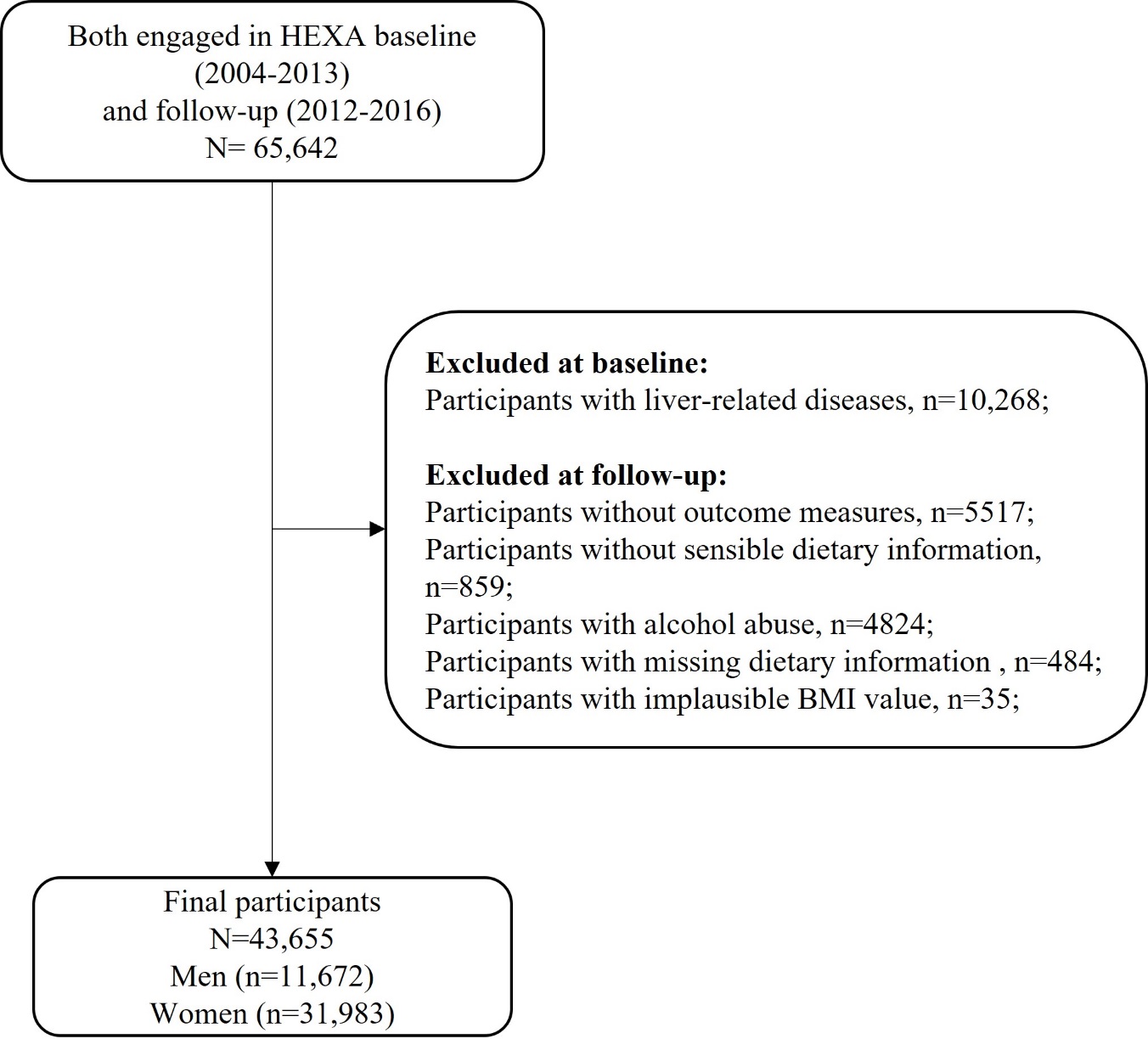


**Supplementary Figure 1.**

Flowchart of participant selection in the current cohort study

BMI, body mass index; HEXA, The Health Examinees study.
